# Incisional Atrial Tachycardia Masquerading As Counter-clockwise Atrial Flutter

**Published:** 2010-03-05

**Authors:** Tchavdar N Shalganov, Mihail M Protich

**Affiliations:** Cardiology Department, National Heart Hospital, Sofia, Bulgaria

**Keywords:** macroreentrant atrial tachycardia, electroanatomic mapping, entrainment

Forty-four-year-old male patient with surgical closure of ostium secundum type atrial septal defect at the age of 9 years had undergone radiofrequency catheter ablation of the cavotricuspid isthmus because of recurrent drug-resistant typical isthmus-dependent atrial flutter, with bi-directional isthmus block achieved. Only few days later he started to suffer again high-rate tachycardia. An atypical atrial flutter was diagnosed on ECG (Figure 1). This one proved to be drug-resistant as well and recurred promptly after cardioversion.

A second ablation was attempted, with 20-polar catheter deployed in the right atrium around the tricuspid annulus such that its tip was in the inferolateral part of the cavotricuspid isthmus, 6-polar catheter in the coronary sinus, and use of EnSite NavX electroanatomic mapping system (St. Jude Medical, Inc., St. Paul, MN, USA). Surprisingly, the intracardiac recordings showed activation sequence resembling the same counter-clockwise atrial flutter that was previously ablated (Figure 2A), although no potentials could be recorded in the ablated area. Pacing the inferolateral isthmus at a rate faster than the tachycardia rate showed concealed entrainment with post-pacing interval only 4 milliseconds longer than the tachycardia cycle length (Figure 2A). However, when entrainment was performed from the septal isthmus, the post-pacing interval was 79 milliseconds longer than the tachycardia cycle length (Figure 2B). No change in the intraatrial activation sequence was induced meaning that the septal isthmus was a bystander site [1].

 Electroanatomic activation map showed macroreentrant atrial tachycardia with the wave-front circulating around the presumed post-operative scar on the right atrial free wall (Figure 3A). The voltage map confirmed the presence of a scar acting as the central obstacle of the reentrant circuit (Figure 3B). A narrow myocardial remnant across the scar (Figure 3B - arrow) served as the isthmus of the circuit. Single radiofrequency application at this site terminated the tachycardia and rendered it uninducible.

Patients with previous congenital heart surgery not infrequently develop atrial tachycardias late after the operation. These tachycardias are predominantly macroreentrant and the cavotricuspid isthmus often is part of the reentrant circuit. The incisional scar in the right atrial free wall and the cavotricuspid isthmus make together long protected corridor potentiating the occurrence and the perpetuation of peritricuspid reentry. Gaps in the scar predispose to the occurrence of reentrant tachycardias in the right atrial free wall with the scar acting as the central obstacle of the circuit [2]. The same substrate can be responsible for the occurrence of dual-loop reentry with one loop almost universally being the peritricuspid circuit of the isthmus-dependent atrial flutter, and the second loop being the scar-dependent circuit in right atrial free wall [2,3].

## Figures and Tables

**Figure 1:**
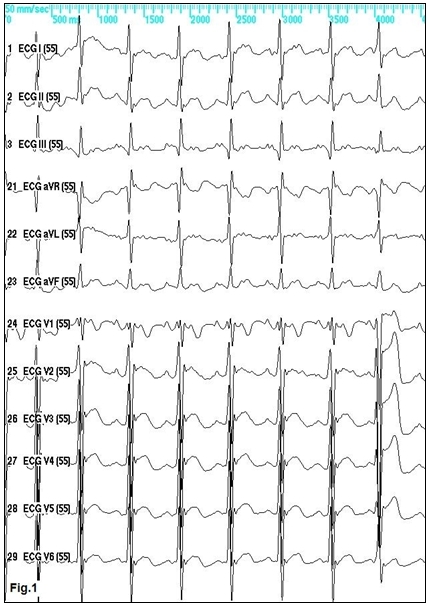
12-lead ECG of the clinical tachycardia

**Figure 2:**
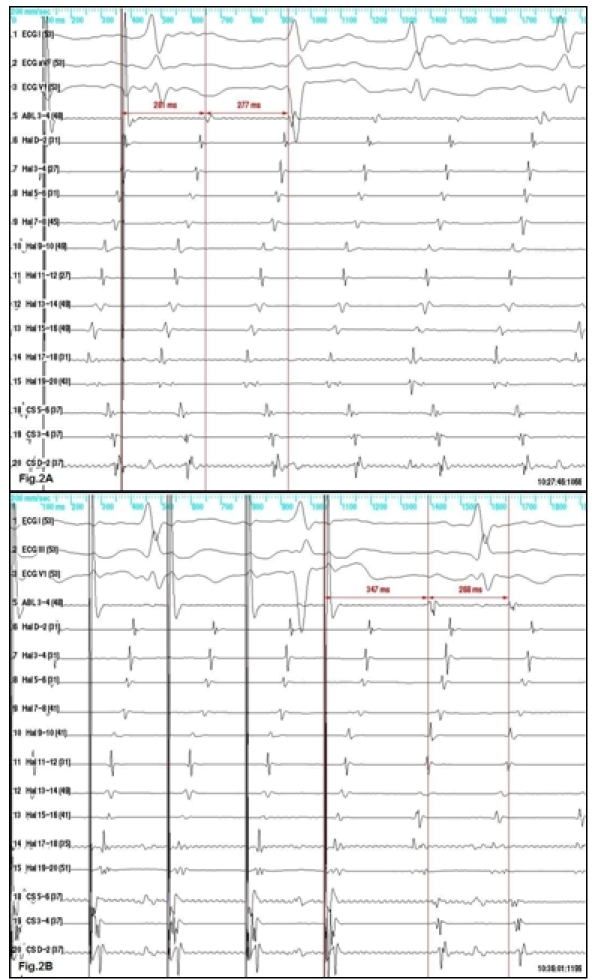
A - pacing the inferolateral isthmus at a cycle length of 260 ms does not alter the activation sequence and the postpacing interval is almost identical to the tachycardia cycle length - concealed entrainment. B - pacing in the septal isthmus at the same pacing rate shows again unaltered activation sequence, but the postpacing interval is very long compared to the tachycardia cycle length - concealed entrainment from a bystander site.

**Figure 3:**
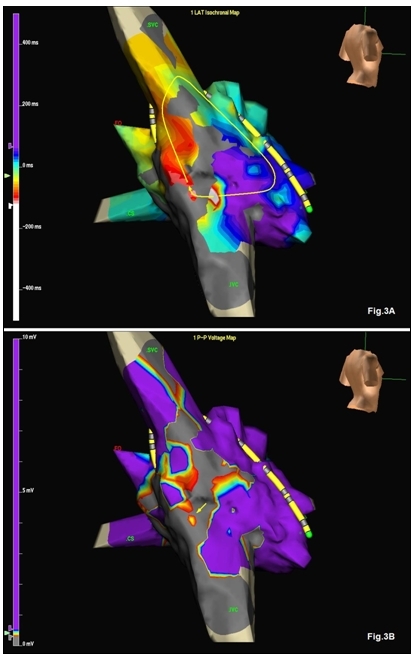
A - activation map of the tachycardia. B - voltage map during tachycardia. The circuit (panel A - curved line and arrow) is located around a scar. The arrow on panel 3B points to the narrow isthmus across the scar.
